# Prevalence of fluoroquinolone resistance and mutations in the *gyr*A, *par*C and *par*E genes of *Riemerella anatipestifer* isolated from ducks in China

**DOI:** 10.1186/s12866-019-1659-4

**Published:** 2019-12-03

**Authors:** Dekang Zhu, Mingyu Zheng, Jinge Xu, Mingshu Wang, Renyong Jia, Shun Chen, Mafeng Liu, Xinxin Zhao, Qiao Yang, Ying Wu, Shaqiu Zhang, Juan Huang, Yunya Liu, Ling Zhang, Yanling Yu, Leichang Pan, Xiaoyue Chen, Anchun Cheng

**Affiliations:** 1 Research Center of Avian Diseases, College of Veterinary Medicine, Sichuan Agricultural University, Chengdu, Sichuan China; 2 Key Laboratory of Animal Disease and Human Health of Sichuan Province, Chengdu, Sichuan China; 3 Institute of Preventive Veterinary Medicine, Sichuan Agricultural University, Chengdu, Sichuan China; 4 Guizhou Animal Husbandry and Veterinary Research Institute, Guiyang, Guizhou China

**Keywords:** *Riemerella anatipestifer*, Fluoroquinolone resistance, Point mutant, *gyr*A gene, *par*C gene, *par*E gene

## Abstract

**Background:**

*Riemerella anatipestifer* is one of the most serious infectious disease-causing pathogens in the duck industry. Drug administration is an important method for prevention and treatment of infection in duck production, leading to widespread drug resistance in *R. anatipestifer*.

**Methods:**

For a total of 162 isolates of *R. anatipestifer,* the MICs were determined for a quinolone antimicrobial agent, namely, nalidixic acid, and three fluoroquinolones, namely, ciprofloxacin, enrofloxacin and ofloxacin. The *gyr*A, *par*C, and *par*E gene fragments were amplified by PCR to identify the mutation sites in these strains. Site-directed mutants with mutations that were detected at a high frequency in vivo were constructed (hereafter referred to as site-directed in vivo mutants), and the MICs of these four drugs for these strains were determined.

**Results:**

In total, 100, 97.8, 99.3 and 97.8% of the 137 *R. anatipestifer* strains isolated between 2013 and 2018 showed resistance to nalidixic acid, ciprofloxacin, enrofloxacin, and ofloxacin, respectively. The high-frequency mutation sites were detected in a total of 162 *R. anatipestifer* strains*,* such as Ser83Ile and Ser83Arg, which are two types of substitution mutations of amino acid 83 in GyrA; Val799Ala and Ile811Val in ParC; and Val357Ile, His358Tyr, and Arg541Lys in ParE. MIC analysis results for the site-directed in vivo mutants showed that the strains with only the Ser83Ile mutation in GyrA exhibited an 8–16-fold increase in MIC values, and all mutants showed resistance to ampicillin and ceftiofur.

**Conclusions:**

The resistance of *R. anatipestifer* to quinolone agents is a serious problem. Amino acid 83 in GyrA is the major target mutation site for the fluoroquinolone resistance mechanism of *R. anatipestifer*.

## Background

*Riemerella anatipestifer* (*R. anatipestifer*) is a nonmotile Gram-negative rod-shaped bacterium that is usually isolated from ducks, geese, and turkeys. *R. anatipestifer* infection can cause pericarditis, peritonitis, fibrinous exudation, diarrhea and neurological symptoms in ducks, which lead to reduced growth rates and high mortality and consequently to great economic loss [1].

Fluoroquinolone antibiotics are one of the most potent broad-spectrum agents commonly used to treat a range of infections [2, 3]. Due to the widespread and indiscriminate use of fluoroquinolone antibiotics, the resistance of *R. anatipestifer* to fluoroquinolones is particularly serious [4, 5].

Many types of antibiotic resistance mechanisms of *R. anatipestifer* have been reported, including those against chloramphenicol [6]; florfenicol [7]; aminoglycosides [8]; macrolides, lincosamides and streptogramin B (collectively referred to as MLS) [9, 10]; and tetracycline [11]. However, few articles on the mechanism of fluoroquinolone resistance associated with *R. anatipestifer* have been published. Resistance to quinolones can occur via multiple mechanisms, including target-site mutations, multidrug resistance (MDR) efflux pumps, changes in membrane permeability, and plasmid-mediated quinolone resistance (PMQR) genes [12].

DNA gyrase and topoisomerase IV can both relax positively supercoiled DNA, albeit with different focuses. DNA gyrase is essential for DNA replication, transcription, and recombination [12]. Topoisomerase IV unlinks newly replicated DNA, thereby allowing chromosome segregation at cell division [13]. Studies on target mutations in quinolone-resistance-determining regions (QRDRs) show that these mutations are the most prevalent mechanism of quinolone resistance. Mutations involved in fluoroquinolone resistance have been extensively studied in other species in recent decades, such as *Escherichia coli* [14], *Mycobacterium tuberculosis* [15], and *Shigella flexneri* [16]; however, research on *R. anatipestifer* is limited. In addition, there may also be major mutations in non-QRDRs [17, 18]. Therefore, in addition to QRDRs, non-QRDRs are also worthy of attention.

Linda [19], Viktòria Làzàr [20] and Csaba Pà [21] et al. all showed that quinolone-resistant *E. coli* mutants exhibited cross-resistance to other types of antibiotics. In addition, Webber et al. [22] reported that DNA gyrase mutants of *Salmonella typhimurium* L821(Ser83Phe) and L825 (Asp87Gly) showed different susceptibilities to some antimicrobials, such as beta-lactams, aminoglycosides, and folate synthesis inhibitors.

In this study, we determined the minimum inhibitory concentration (MIC) values of four quinolone drugs for 162 *R. anatipestifer* isolates to investigate the prevalence of quinolone resistance in *R. anatipestifer*. Then, we detected mutations in the *gyr*A, *par*C and *par*E gene fragments of the 162 *R. anatipestifer* isolates and constructed *R. anatipestifer* mutants with high-frequency in vivo mutation sites to explore whether mutations at these sites contribute to the quinolone resistance mechanism of *R. anatipestifer* and to investigate whether these mutations can cause cross-resistance.

## Methods

### *R. anatipestifer* strains

A total of 162 *R. anatipestifer* strains were used in this study. Among these strains, 137 clinical isolates for fluoroquinolone resistance analysis were obtained from 48 duck farms from 2013 to 2018, 15 other clinical isolates were isolated from 14 duck farms from 1998 to 2012, one strain was obtained from the American Type Culture Collection (ATCC) and 9 strains were obtained from the Culture Collection of the University of Gothenburg (CCUG). Detailed strain information is shown in Additional file 1: Table S1.

All *R. anatipestifer* isolates were cultured on 5% blood tryptic soy agar (TSA; Oxoid, Basingstoke, UK). These inoculated plates were incubated at 37 °C in a 5% CO_2_ atmosphere for 24 h. Polymerase chain reaction (PCR) amplification of 16S rRNA was performed to identify *R. anatipestifer* as described previously [9].

### Minimum inhibitory concentrations (MICs) of quinolones

MICs of nalidixic acid (Aladdin, USA), ciprofloxacin (Aladdin, USA), enrofloxacin (Aladdin, USA) and ofloxacin (Meilunbio, China) for 162 *R. anatipestifer* isolates were determined using the standard microscale broth dilution method, performed according to Clinical and Laboratory Standard Institute (CLSI) criteria [23], to analyze the antimicrobial susceptibility of randomly collected isolates between 1932 and 2018.

To explore whether the *R. anatipestifer* site-directed in vivo mutants showed cross-resistance phenotypes with other nonquinolone antibiotics, the MICs of several other nonquinolone antibiotics such as ampicillin (Aladdin, USA), ceftiofur (Meilunbio, China), amikacin (Meilunbio, China), florfenicol (Aladdin, USA), doxycycline (Meilunbio, China) and lincomycin (Aladdin, USA) were determined for these strains.

The concentration of these antimicrobial agents in 96-well plates ranged from 0.25 to 1024 μg/mL, except that of nalidixic acid, which ranged from 0.5 to 2048 μg/mL. *E. coli* ATCC 25922 was used for quality control. The experiments were repeated in triplicate.

### PCR amplification of the *gyr*A, *par*C and *par*E genes

Thirty-seven complete genomes of *R. anatipestifer* strains were sequenced by our team in a previous study, and these sequences were uploaded to the National Center for Biotechnology Information (NCBI, http://www.ncbi.nlm.nih.gov/genome) database. The accession numbers of these complete genomes are listed in Additional file 2: Table S2. We compared the *gyr*A, *gyr*B, *par*C, and *par*E genes of these thirty-seven complete genomes. The comparison results are shown in Additional file 3-6: Table S3-S6. A region with a high mutation probability that included a large number of mutations was selected as the target region for PCR amplification. The region includes more than just the QRDR. Five fragments were selected (GyrA amino acids 51–327 and 442–726; ParC amino acids 349–615 and 610–857; and ParE amino acids 312–590). There were no eligible areas in the GyrB. The primers were designed with ATCC 11845 as the template to amplify the above five sequences. In addition to the 37 whole-genome sequencing strains, the remaining 125 clinical isolates were prepared as DNA templates by heat and lysis. The above five regions of 125 clinical isolates were amplified to obtain the mutant sites in these regions. In addition, Sanger sequencing was also carried out for the above five regions of the 37 whole-genome sequencing strains to verify the accuracy of the sequences. PCR primers are listed in Additional file 7: Table S7.

### Sequence alignment analysis

The sequenced fragments of the 125 clinical isolates and the 37 complete genomes of *R. anatipestifer* strains were compared with the genome of ATCC 11845 (as a reference strain) by BLAST [24]. The genomic sequence of ATCC 11845 was downloaded from NCBI. MEGA 7 [25] was used for alignment and to obtain mutation sites in all *R. anatipestifer* strains.

### Construction of *R. anatipestifer* mutants in vivo

The regions where the high-frequency mutation sites were located were selected to design primers. The target fragments of the three genes of ATCC 11845 were amplified via PCR, and the amplified fragments were ligated to a *Nco*I/*Xho*I-digested pOES suicide vector to generate pOES-fragments (*gyr*A fragment-1, 514 bp; *gyr*A fragment-2, 944 bp; *par*C fragment-1, 454 bp; *par*C fragment-2, 433 bp; *par*E fragment, 1026 bp). Subsequently, pOES fragments were transformed into *E. coli* DH5α. The recombinant plasmids were extracted using the E.Z.N.A® Plasmid Mini Kit I (Omega Bio-Tek, USA). The QuikChange® Lightning Site-Directed Mutagenesis Kit (Stratagene, CA) was used to construct the site-directed mutations in recombinant plasmids. Subsequently, these recombinant plasmids containing site-directed mutations were transformed into *E. coli* S17–1. The transconjugants were selected on LB plates with ampicillin (Amp, 100 μg/mL).

The donor strain *E. coli* S17–1 harboring the suicide vector pOES fragments and the recipient strain *R. anatipestifer* ATCC 11845 were grown on LB and sheep blood plates, respectively, at 37 °C overnight. Targeted mutants were constructed by introducing recombinant DNA into the cells by conjugation [26]. The transconjugants were selected on blood agar plates supplemented with cefoxitin (Cfx, 1 μg/mL) and kanamycin (Kana, 50 μg/mL). Transconjugants were shaken in GC broth (GCB) at 37 °C overnight to lose the suicide plasmid. Then, 200 μL of the appropriate dilution was plated on GCB agar supplemented with 13 mM p-Cl-Phe to select clones without the plasmid [27]. Clones were screened by PCR using primers cfx P1/P2 to choose Cfx-sensitive colonies. Then, the Cfx-sensitive colonies were sequenced with the corresponding primers. The primers and plasmids used in this study are shown in Table 1. Growth curves were plotted for the ATCC 11845 strain and *R. anatipestifer* mutants grown in TSB medium at 37 °C. OD_600_ was recorded at 2-h intervals.

**Table 1:** Antibiotic resistance phenotypes of *R. anatipestifer* strains collected between 2013 and 2018

Years	Antibiotics	MICs (μg/mL) and proportion of strains (%)										
		0.5	2	4	8	16	32	64	128	256	512	1024
2013 *n* = 13	NA									7.69	61.5	30.8
	CIP	7.69				7.69	84.6					
	ENR	7.69				7.69	7.69	53.8	23.1			
	OFX		7.69				46.2	23.1	23.1			
2014~2015 *n* = 6	NA									16.7	83.3	
	CIP			16.7		16.7	50	16.7				
	ENR			16.7			16.7	50	16.7			
	OFX			16.7		16.7	33.3	16.7	16.7			
2016 *n* = 18	NA									11.1	77.8	11.1
	CIP				5.6	55.6	38.9					
	ENR				5.6	22.2	27.8	33.3	11.1			
	OFX				11.1	44.4	44.4					
2017 *n* = 60	NA									10	42	8
	CIP		3.3	5.0	1.7	21.7	61.7	6.7				
	ENR			8.3	1.7	8.3	28.3	36.7	16.7			
	OFX		3.3	5.0	11.7	28.3	20	18.3	13.3			
2018 *n* = 40	NA									17.5	57.5	25
	CIP				7.5	35	35	22.5				
	ENR				2.5	50	10	12.5	25			
	OFX				2.5	47.5	12.5	22.5	15			

^a^*NA* nalidixic acid; *CIP* ciprofloxacin; *ENR* enrofloxacin; *OFX* ofloxacin

## Results

### Minimum inhibitory concentrations (MICs) of nalidixic acid, ciprofloxacin, enrofloxacin, and ofloxacin

The MICs of four antibacterial drugs were determined for a total of 162 *R. anatipestifer* strains. The MIC values for all strains are shown in Additional file 1: Table S1.

Since the number of collected strains before 2013 was limited, we selected 137 *R. anatipestifer* strains from 48 farms from 2013 to 2018 to analyze the prevalence of quinolone resistance (Table 1). The MIC values of four quinolone drugs for the 137 *R. anatipestifer* strains were all greater than 90%. A total of 100% (137/137) of the *R. anatipestifer* strains were resistant to nalidixic acid*.* Meanwhile, *R. anatipestifer* strains that showed resistance to ciprofloxacin, enrofloxacin, or ofloxacin were determined to be 97.8% (134/137), 99.3% (136/137) or 97.8% (134/137) of the total, respectively.

### Amino acid substitutions of GyrA, GyrB and ParC in quinolone-resistant *R. anatipestifer*

We sequenced five gene fragments of *gyr*A, *par*C and *par*E of 162 *R. anatipestifer* strains. The detailed amino acid substitutions in the amplified fragments are shown in Additional file 1: Table S1. In GyrA, amino acid position 83 contained two mutation types: Ser83Arg (26/162) and Ser83Ile (125/162). Moreover, the strains that were resistant to ciprofloxacin (149/162), enrofloxacin (151/162), and ofloxacin (149/162) all had mutations at position 83. In addition, the mutation frequencies of Cys465Arg (69/162) in GyrA; Val586Thr/Ala (67/162), Val799Ala (117/162), and Ile811Val (109/162) in ParC; and Val357Ile (131/162), His358Tyr (131/162), Arg541Lys (133/162), and Asp564Lys (72/162) in ParE were all higher than 40%; these sites were defined as high-frequency mutation sites. Asn202Glu (52/162) in GyrA and Ile390Thr (45/162), Gly752Val (42/162), Val768Ala (42/162), Glu827Asp (42/162), and Met833Ile (42/162) in ParC had mutation frequencies between 20 and 40%; these sites were defined as medium-frequency mutation sites. Twenty-eight amino acid mutations with mutation frequencies lower than 20% were observed (the detailed mutation sites are shown in Additional file 1: Table S1).

The correspondence between different mutation types and MIC values of different drugs are shown in Additional file 8: Table S8. The number of mutants containing Ser83Ile in GyrA; Val799Ala and Ile811Val in ParC; and Val357Ile, His358Tyr, and Arg541Lys in ParE was the highest (44/162), whereas mutants with Ser83Ile in GyrA and Val357Ile, His358Tyr and Arg541Lys in ParE were the second most frequent (21/162, 13.0%). Compared to the ATCC 11845 strain, the strains with these mutation types exhibited a 32- to 512-fold increase in the MICs of the fluoroquinolone drugs, but the MIC value of nalidixic acid did not change significantly.

### Characterization of *R. anatipestifer* mutants

Eight mutant strains, which were selected based on sites with mutation frequencies greater than 40%, were constructed to explore the contribution of different mutation sites to the resistance of *R. anatipestifer*. The MIC values of four antibacterial drugs were determined for the mutants (Table 2). Regardless of the amino acid substitution, the MIC of nalidixic acid did not change significantly. The high-frequency mutations Val799Ala and Ile811Val in ParC and/or Val357Ile, His358Tyr and Arg541Lys in ParE, which were present without the amino acid 83 substitution in GyrA, had no significant effect on MIC values. Compared to the parent strain, only the mutant strains with the amino acid 83 substitution in GyrA, namely, the ATCC *gyrA* (Ser83Ile) and ATCC *gyrA* (Ser83Ile) + *par*E (Val357Ile, His358Tyr, Arg541Lys) strains, exhibited an 8- to 16-fold enhancement in resistance to fluoroquinolones. In contrast, the high-frequency mutation sites Cys465Arg in GyrA; Val799Ala and Ile811Val in ParC; and Val357Ile, His358Tyr and Arg541Lys in ParE had no effect on quinolone resistance.

**Table 2:** MICs for site-directed *R. anatipestifer* mutants

Mutants	MICs (μg/mL)^a^									
	NA	CIP	ENR	OFX	AMP	EFT	AK	FFC	DO	MY
ATCC 11845	256	0.25	0.25	0.25	< 0.25	1	64	2	< 0.25	0.25
ATCC *gyr*A (Ser83Ile)	512	2	4	4	64	128	128	2	< 0.25	0.25
ATCC *gyr*A (Cys465Arg)	256	0.25	< 0.25	0.25	16	32	32	2	< 0.25	< 0.25
ATCC *par*C (Val586Ala)	256	0.25	< 0.25	0.5	64	128	64	2	< 0.25	< 0.25
ATCC *par*C (Ile811Val)	256	< 0.25	< 0.25	0.25	64	128	64	2	< 0.25	0.25
ATCC *par*C (Val799Ala, Ile811Val)	512	0.25	0.25	0.25	64	128	64	2	< 0.25	0.25
ATCC *par*E (Val357Ile, His358Tyr)	256	0.25	0.25	0.25	64	128	64	2	< 0.25	0.25
ATCC *par*E (Val357Ile, His358Tyr, Arg541Lys)	512	0.25	0.25	0.25	32	128	64	2	< 0.25	0.25
ATCC *gyr*A (Ser83Ile) + *par*E (Val357Ile, His358Tyr, Arg541Lys)	256	4	4	2	128	256	64	2	< 0.25	< 0.25

^a^*NA* nalidixic acid; *CIP* ciprofloxacin; *ENR* enrofloxacin; *OFX* ofloxacin; *AMP* ampicillin, *EFT* ceftiofur; *AK* amikacin; *FFC* florfenicol; *DO* doxycycline; *MY* lincomycin

To understand whether the site-directed mutants exhibit low sensitivity to other types of antibiotics, the MICs of six nonquinolone antibiotics were determined for these strains. The results showed that the MICs of ampicillin and ceftiofur were significantly increased for the mutants. Interestingly, regardless of which gene among *gyr*A, *par*C, and *par*E has amino acid substitutions, the MIC value of ampicillin and ceftiofur increased significantly.

**Fig. 1:**
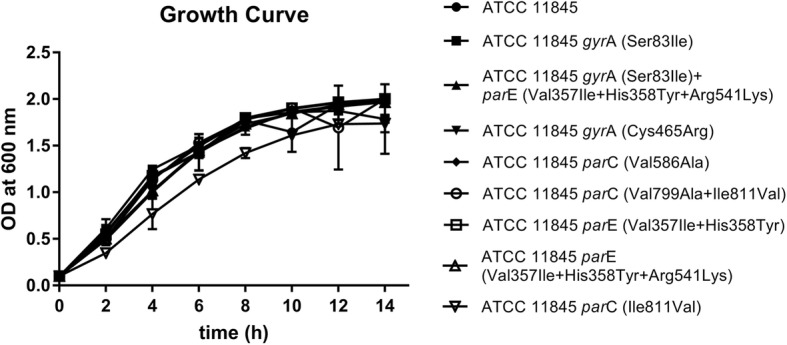
Growth curves for *R. anatipestifer* ATCC 11845 and *R. anatipestifer* site-directed mutants. *R. anatipestifer* ATCC 11845 and site-directed mutants were cultured (OD_600_ = 0.1) in 20 mL of TSB, and the growth curves were determined

To explore whether these site mutations would affect the adaptability of the strain, the growth curves of the site-directed mutant strains and the parent strain were plotted (Fig. 1). Compared to the parent strain, the mutants exhibited no significant difference in growth, except for the ATCC *par*C (Ile811Val) strain.

## Discussion

In the past six years, the resistance rate of *R. anatipestifer* to four quinolones has increased. The resistance rate to nalidixic acid, which was 60% before 2003 [28] and 87.4% between 1999 and 2009 [29], has risen to 100% between 2013 and 2018. The resistance rate to ciprofloxacin increased from 23.1 to 59.2% between 1998 and 2010 [29–31]. The resistance rate to enrofloxacin was approximately 60% between 1999 and 2010 [29, 30]. Meanwhile, Zhong et al. [31] and Zhang et al. [29] determined that the resistance rate of *R. anatipestifer* isolated from different years to ofloxacin was approximately 24%. Nevertheless, the resistance rate to ofloxacin increased to 51.5% between 2008 and 2010. The resistance rates of *R. anatipestifer* isolated between 2013 and 2018 to CIP, ENR and OFX were 97.8, 99.3 and 97.8%, respectively. Compared with the resistance rate determined in the past, it can be seen that the resistance of *R. anatipestifer* to quinolones has increased. The high prevalence of fluoroquinolone resistance in *R. anatipestifer* isolates from ducks may be due to the overuse and abuse of fluoroquinolones in duck disease treatment.

The MIC of nalidixic acid is interesting. The MIC values of nalidixic acid against ATCC 11845 and CCUG 18373, which were isolated in 1932 and 1955, respectively, were both 256 μg/mL, notwithstanding the fact that quinolones were not used in that period. Therefore, in addition to point mutations, there are other mechanisms that mediate resistance to nalidixic acid.

The emergence of mutations is consistent with the history of the development of quinolone drugs. In the 1980s, third-generation quinolone antibiotics were synthesized successively. The strains isolated from 1960 to 1980 are sensitive to FQs, while the RA-CH-2 and RCAD0392 strains isolated in the 1990s showed a resistant phenotype to FQs (Additional file 1: Table S1). The proportion of fluoroquinolone-resistant strains increased significantly. This phenomenon may be due to the increased use of FQs and/or the third-generation quinolone antibiotics being highly conducive to enriching *R. anatipestifer* resistant strains.

The presence of the amino acid 83 mutation increases the MIC value of fluoroquinolones. All fluoroquinolone-resistant isolates had a mutation at the amino acid 83 site in GyrA (Additional file 1: Table S1), and the site-directed mutants with an amino acid 83 mutation showed an 8- to 16-fold increase in MIC values. These results indicate that the amino acid 83 mutation in GyrA is critical to confer fluoroquinolone resistance to *R. anatipestifer*. This finding is consistent with previous research results [32, 33]. There are two mutant types at the amino acid 83 position in GyrA, Ser83Arg and Ser83Ile. Previous reports suggest that the mutation type Ser83Ile provides more resistance to FQs than Ser83Arg [29]. However, we found that several strains containing the Ser83Arg mutation type have FQ MICs ranging from 64 to 128 μg/mL. Both Ser83Ile and Ser83Arg can confer low-level or high-level resistance to strains, and Ser83Arg can also confer sensitivity to strains. The same type of mutation also has a variety of base substitution methods. The Ser83Ile group contains AGC-ATC and AGC-ATT mutations, while the Ser83Arg group contains AGC-AGA and AGC-CGC mutations. The same mutant types with different base substitutions had difference in resistance to FQs. For strains with Ser83Arg, some of which had the AGC-AGA base substitution type, the MICs of FQs were 4 μg/mL or less; the strains in which the base substitution type was AGC-CGC had MIC values of FQs greater than or equal to 8 μg/mL. The reason for this phenomenon remains to be explored.

It is strange that while more than 90% of *R. anatipestifer* strains have high-frequency mutations in *par*C and *par*E, these mutations have no significant effect on fluoroquinolone resistance. Perhaps these types of high-frequency mutations are highly adaptive to the evolution of the strain [12, 34]; however, the exact significance of these mutations remains to be explored. Interestingly, although these high-frequency mutations have no significant effect on fluoroquinolone resistance, they all increase the resistance of strains to beta-lactams. This result is consistent with previous reports. The reason for the change in the sensitivity to nonquinolones is probably due to altered patterns of supercoiling and hence global expression of stress response pathways [20, 22].

The RCAD0133 strain has 22 mutations, including all high-frequency mutations; however, this strain has a low level of resistance to fluoroquinolone drugs. These results suggest that not all mutations confer resistance to fluoroquinolones [35] and that the number of mutations is not related to the level of fluoroquinolone resistance.

The substitutions Asp87His in GyrA and Arg120Glu in ParC, which might be associated with fluoroquinolone resistance, were reported by Sun et al. [5]. Additionally, Zhang et al. [29] suggested that Gly81Asp in GyrA might play a sufficient role in enrofloxacin resistance. However, none of these three mutation sites were detected in this test. The Glu202Asn mutation in GyrA [29] and mutations at positions 357 and 358 in ParE, which may increase the resistance of RA to fluoroquinolones, were reported. These mutation sites are consistent with the detected mutations in this study, but the MIC results for the site-directed mutants showed that amino acids 357 and 358 had no significant effect on fluoroquinolone resistance. ATCC *par*C (Val799Ala, Ile811Val) grew significantly better than ATCC *par*C (Ile811Val) in the same media conditions (Fig. 1). This phenomenon may be due to the generation of compensatory mutations that increase the adaptability of the strain [36].

There are many strains that have the same mutation type but have different drug resistance phenotypes, such as RCAD0354 and RCAD0356, but these strains exhibit a 2- to 8-fold difference in MICs of FQs. This phenomenon indicates that there may be other mechanisms, such as other low-frequency mutations, undetected sites or efflux pumps.

## Conclusions

The resistance rate of *R. anatipestifer* to four quinolones is considerably high in the duck industry. Sequencing results and the MIC of site-directed mutants showed that only the strains containing the amino acid 83 mutation exhibited resistance to FQs; the other high-frequency mutation sites did not significantly contribute to the MICs. This study highlights the importance of using fluoroquinolones reasonably and correctly to reduce the emergence of multidrug-resistant strains; moreover, it provides data for the molecular detection of fluoroquinolone-resistant *R. anatipestifer* strains.

## Supplementary information

**Additional file 1: Table S1.** Information on the mutation sites in the *gyr*A, *par*C and *par*E genes of *R. anatipestifer*

**Additional file 2: Table S2.** Origin and accession numbers of 37 whole-genome sequences of *R. anatipestifer*

**Additional file 3: Table S3.** Amino acid sequence alignment of GyrA from the 37 whole-genome-sequenced *R. anatipestifer* strains

**Additional file 4: Table S4.** Amino acid sequence alignment of GyrB from the 37 whole-genome-sequenced *R. anatipestifer* strains

**Additional file 5: Table S5.** Amino acid sequence alignment of ParC from the 37 whole-genome-sequenced *R. anatipestifer* strains

**Additional file 6: Table S6.** Amino acid sequence alignment of ParE from the 37 whole-genome-sequenced *R. anatipestifer* strains

**Additional file 7: Table S7.** Primers and plasmids used in this study

**Additional file 8: Table S8.** Antibiotic phenotype and amino acid substitutions in GyrA, ParC and ParE of *R. anatipestifer*.

## Data Availability

The data supporting the results of this paper are available in the NCBI WGS database. The accession numbers of *R. anatipestifer* genomes are included in Additional file 2: Table S2.

## Abbreviations

ATCC: American Type Culture Collection
CCUG: Culture Collection of the University of Gothenburg
CLSI: Clinical and Laboratory Standards Institute
MDR: multidrug resistance
MIC: minimum inhibitory concentration
NCBI: National Center for Biotechnology Information
PCR: polymerase chain reaction
PMQR: plasmid-mediated quinolone resistance
QPDRs: quinolone-resistance-determining regions
TSA: tryptone soy agar
TSB: tryptone soy broth
