# Supplementary material for: Prevalence of fluoroquinolone resistance and mutations in the gyrA, parC and parE genes of Riemerella anatipestifer isolated from ducks in China
Source: BMC Microbiol. 2019 Dec 3;19:271. doi: 10.1186/s12866-019-1659-4 (PMC6892153; doi:10.1186/s12866-019-1659-4)
Supplement: Supplementary file 2 — Additional file 2: Table S2. Origin and accession numbers of 37 whole-genome sequences of R. anatipestifer [file 12866_2019_1659_MOESM2_ESM.xls]

**Additional Table S2. The origin and accession number of 37 whole genome sequence of *R. anatipestifer***

| Name | Isolated date | Isolated location | Host | Length (bp) | Accession number |
| --- | --- | --- | --- | --- | --- |
| ATCC 11845 | 1932 | USA | Duck | 2164087 | CP003388 |
| CCUG18373 | 1955 | USA | Duck | 2149492 | QXHN00000000 |
| CCUG25001 | 1966-1969 | UK | Duck | 2299422 | QXHO00000000 |
| CCUG25002 | 1976 | UK | Duck | 2332779 | QXHP00000000 |
| CCUG25004 | 1976 | UK | Duck | 2139715 | QXHQ00000000 |
| CCUG25005 | 1966-1969 | UK | Duck | 2167466 | QXHR00000000 |
| CCUG25010 | 1976 | UK | Duck | 2414224 | QXHT00000000 |
| CCUG25054 | 1976 | UK | Duck | 2308571 | QXHU00000000 |
| CCUG25008 | 1966-1969 | UK | Duck | 2291094 | QXHS00000000 |
| CCUG25055 | 1976 | USA | Duck | 2265911 | QXHV00000000 |
| RA-CH-1 | 1993 | China | Duck | 2309519 | CP003787 |
| RA-CH-2 | 1996 | China | Duck | 2166321 | CP004020 |
| RCAD0111 | 2012 | China | Duck | 2362938 | LUDR00000000 |
| RCAD0121 | 2011 | China | Duck | 2216702 | LUDI00000000 |
| RCAD0122 | 2012 | China | Duck | 2205797 | LUDU00000000 |
| RCAD0123 | 2011 | China | Duck | 2086211 | LUDP00000000 |
| RCAD0124 | 2011 | China | Duck | 2198894 | LUDQ00000000 |
| RCAD0125 | 2012 | China | Duck | 2143994 | LUDJ00000000 |
| RCAD0127 | 2013 | China | Duck | 2093347 | LUDV00000000 |
| RCAD0131 | 2011 | China | Duck | 2110744 | LUDS00000000 |
| RCAD0133 | 2016 | China | Duck | 2509140 | CP029760 |
| RCAD0134 | 2011 | China | Duck | 2158756 | LUDO00000000 |
| RCAD0142 | 2011 | China | Duck | 2109496 | LUDG00000000 |
| RCAD0147 | 2011 | China | Duck | 2098201 | LUDN00000000 |
| RCAD0150 | 2011 | China | Duck | 2098894 | LUDM00000000 |
| RCAD0181 | 2014 | China | Duck | 2115418 | LUDL00000000 |
| RCAD0183 | 2014 | China | Duck | 2204034 | LUDK00000000 |
| RCAD0188 | 2014 | China | Duck | 2162414 | LUDH00000000 |
| RCAD0135 | 2017 | China | Duck | 2286048 | QXQO00000000 |
| RCAD0282 | 2014 | China | Duck | 2285175 | QXQL00000000 |
| RCAD0152-1 | 2011 | China | Duck | 2236324 | QXQN00000000 |
| RCAD0179 | 2013 | China | Duck | 2205109 | QXQM00000000 |
| RCAD0377 | 2017 | China | Duck | 2094374 | QXQK00000000 |
| RCAD0414 | 2017 | China | Duck | 2356504 | QXFQ00000000 |
| RCAD0421 | 2017 | China | Duck | 2120678 | QXQJ00000000 |
| RCAD0422 | 2017 | China | Duck | 2119439 | QXQI00000000 |
| RCAD0427 | 2017 | China | Duck | 2261483 | QXQH00000000 |
