# Supplementary material for: Prevalence of fluoroquinolone resistance and mutations in the gyrA, parC and parE genes of Riemerella anatipestifer isolated from ducks in China
Source: BMC Microbiol. 2019 Dec 3;19:271. doi: 10.1186/s12866-019-1659-4 (PMC6892153; doi:10.1186/s12866-019-1659-4)
Supplement: Supplementary file 7 — Additional file 7: Table S7. Primers and plasmids used in this study [file 12866_2019_1659_MOESM7_ESM.docx]

**Additional Table S7.** Primers and plasmids used in this study.

| **Primer or plasmid** | **Description** | **Source or reference** |
| --- | --- | --- |
| Primers for amplifying *gyr*A, *par*C and *par*E gene high mutation frequency regions | |  |
| *gyr*A-RD-P1 | 5′ GGTATGTATGGATTGGGAG 3′ | This study |
| *gyr*A-RD-P2 | 5′ CGAAGTTTGTAACGCCG 3′ | This study |
| *gyr*A-RD2-P1 | 5′ CCGTGATGAGTACAAAGC 3′ | This study |
| *gyr*A-RD2-P2 | 5′ CCTCTGAATTTCCTTCAGAC 3′ | This study |
| *par*C-RD-P1 | 5′ GACAAGTGGCATTTTGC 3′ | This study |
| *par*C-RD-P2 | 5′ CGTATTCTAGCATTAGGC 3′ | This study |
| *par*C-RD2-P1 | 5′ GAAGCCTAATGCTAGAATAC 3′ | This study |
| *par*C-RD2-P2 | 5′ ACCTCCAAATAAATCTCC 3′ | This study |
| *par*E-RD-P1 | 5′ GAATCTCAAACCAAAACC 3′ | This study |
| *par*E-RD-P2 | 5′ GAATTTGGTCTATAGTTTGG 3′ | This study |
| Primers for cloning the *gyr*A, *par*C and *par*E gene fragments^a^ | |  |
| *gyr*A-PD-F | 5′ CATGCCATGGGTGGACGAAATGAAGTCC 3′ | This study |
| *gyr*A-PD-R | 5′ CCGCTCGAGGAGGCGCCATATTAGTTG 3′ | This study |
| *gyr*A-PD-F2 | 5′ CATGCCATGGGGCGCCTCATAACTTATC 3′ | This study |
| *gyr*A-PD-R2 | 5′ CCGCTCGAGTGGACATTTCACCTCCAG 3′ | This study |
| *par*C-PD-F | 5′ CATGCCATGGTCGCTTTGATGATGAGT 3′ | This study |
| *par*C-PD-R | 5′ CCGCTCGAGTTAACCTCCAAATAAATC 3′ | This study |
| *par*C-PD-F2 | 5′ CATGCCATGGGTGTTTGATTGCTCCG 3′ | This study |
| *par*C-PD-R2 | 5′ CCGCTCGAGCTACCTTTTTTACGGCG 3′ | This study |
| *par*E-PD-F | 5′ CATGCCATGGGGACAAAATACCACACAAG 3′ | This study |
| *par*E-PD-R | 5′ CCGCTCGAGACTGCTGTCTATCAGGTG 3′ | This study |
| Primers for site-directed mutagenesis^b^ | |  |
| *gyr*A-83-F | 5′ CACCCACATGGTGATAtCTCCGTTTATGACGC 3′ | This study |
| *gyr*A-83-R | 5′ GCGTCATAAACGGAGaTATCACCATGTGGGTG 3′ | This study |
| *gyr*A-465-F | 5′ GCTAACGAAGCTAGAcGCTACCAAATCATTAAAG 3′ | This study |
| *gyr*A-465-R | 5′ CTTTAATGATTTGGTAGCgTCTAGCTTCGTTAGC 3′ | This study |
| *par*C-586-F | 5′ CCTTTAGCTTCAGAAGcTAAAGGTTCAGAAGTGC 3′ | This study |
| *par*C-586-R | 5′ GCACTTCTGAACCTTTAgCTTCTGAAGCTAAAGG 3′ | This study |
| *par*C-799-F | 5′ GATATAGACGAATTTATAGcAGTTAAAGGGATTAAAGC  AATGGG 3′ | This study |
| *par*C-799-R | 5′ CCCATTGCTTTAATCCCTTTAACTgCTATAAATTCGTCTAT  ATC 3′ | This study |
| *par*C-811-F | 5′ GCAATGGGGAATCAGTTTgTTAAGGAAAAAGTGAAATCC 3′ | This study |
| *par*C-811-R | 5′ GGATTTCACTTTTTCCTTAAcAAACTGATTCCCCATTGC 3′ | This study |
| *par*E-357-F | 5′ GAGGTTGCAGAAGCGaTTCATAAGAAAATTTTGG 3′ | This study |
| *par*E-357-R | 5′ CCAAAATTTTCTTATGAAtCGCTTCTGCAACCTC 3′ | This study |
| *par*E-358-F | 5′ GAGGTTGCAGAAGCGATTtATAAGAAAATTTTGG 3′ | This study |
| *par*E-358-R | 5′ CCAAAATTTTCTTATaAATCGCTTCTGCAACCTC 3′ | This study |
| Primers for identification of *R. anatipestifer* *in vivo* mutations | |  |
| cfx-P1 | 5′ CTTGGAGTCCTATGCTG 3′ | This study |
| cfx-P2 | 5′ CGCTATAACAACCCCTTC 3′ | This study |
| 16s rRNA F | 5′ CTTCGGATACTTGAGAGCG 3′ | This study |
| 16s rRNA R | 5′ GCAGCACCTTGAAAATTGT 3′ | This study |
| Plasmids |  |  |
| pOES | Suicide plasmid carrying *EXphe*S*, AmpR (CfxR) | Liu et al. [27] |

^a^Underline indicates the restriction sites. CCATGG is the *Nco*I site, while CTCGAG is the *Xho*I site.

^b^ Lowercase letters indicate mutated bases.
