# Supplementary material for: Prevalence of fluoroquinolone resistance and mutations in the gyrA, parC and parE genes of Riemerella anatipestifer isolated from ducks in China
Source: BMC Microbiol. 2019 Dec 3;19:271. doi: 10.1186/s12866-019-1659-4 (PMC6892153; doi:10.1186/s12866-019-1659-4)
Supplement: Supplementary file 8 — Additional file 8: Table S8. Antibiotic phenotype and amino acid substitutions in GyrA, ParC and ParE of R. anatipestifer. [file 12866_2019_1659_MOESM8_ESM.docx]

**Additional Table S8.** Antibiotic phenotype and amino acid substitutions in GyrA, ParC and ParE of *R. anatipestifer*

| sum  isolate no. | MIC range of quinolone (μg/mL)^a^ | | | | each type isolate no. | amino acid substitutions(frequency>20%) | | |
| --- | --- | --- | --- | --- | --- | --- | --- | --- |
|  | NA | CIP | ENR | OFX |  | GyrA | ParC | ParE |
| 3 | 256 | 0.25 | 0.25 | <0.25 | 1 |  |  |  |
|  | 256 | 0.25 | 0.25 | 0.25 | 1 |  |  |  |
|  | 256 | 0.5 | 0.5 | 2 | 1 |  |  |  |
| 1 | 64 | <0.25 | <0.25 | <0.25 | 1 |  | Ile390Thr, Val586Ala, Gly752Val, Val768Ala, Val799Ala, Ile811Val, Glu827Asp, Met833Il | Val357Ile, His358Tyr, Arg541Lys, Asp564Lys |
| 1 | 8 | <0.25 | <0.25 | <0.25 | 1 | Asn202Glu, Cys465Arg | Val586Ala | Arg541Lys, Asp564Lys |
| 6 | 8 | <0.25 | <0.25 | <0.25 | 2 | Asn202Glu, Cys465Arg | Ile390Thr, Val586Ala, Gly752Val, Val768Ala, Val799Ala, Ile811Val, Glu827Asp, Met833Il | Val357Ile, His358Tyr, Arg541Lys, Asp564Lys |
|  | 64 | <0.25 | <0.25 | <0.25 | 1 |  |  |  |
|  | 32 | <0.25 | <0.25 | <0.25 | 2 |  |  |  |
|  | 32 | <0.25 | <0.25 | 0.25 | 1 |  |  |  |
| 1 | 512 | 32 | 64 | 32 | 1 | Ser83Ile | - | - |
| 21 | 256 | 16 | 32 | 32 | 1 | Ser83Ile | - | Val357Ile, His358Tyr, Arg541Lys |
|  | 256 | 16 | 64 | 32 | 1 |  |  |  |
|  | 256 | 32 | 64 | 32 | 1 |  |  |  |
|  | 512 | 16 | 16 | 16 | 1 |  |  |  |
|  | 512 | 16 | 32 | 16 | 1 |  |  |  |
|  | 512 | 16 | 32 | 32 | 1 |  |  |  |
|  | 512 | 16 | 64 | 32 | 1 |  |  |  |
|  | 512 | 32 | 32 | 32 | 2 |  |  |  |
|  | 512 | 32 | 64 | 32 | 3 |  |  |  |
|  | 512 | 32 | 64 | 64 | 2 |  |  |  |
|  | 512 | 32 | 128 | 32 | 1 |  |  |  |
|  | 512 | 32 | 128 | 64 | 1 |  |  |  |
|  | 512 | 32 | 128 | 128 | 2 |  |  |  |
|  | 512 | 64 | 128 | 64 | 1 |  |  |  |
|  | 1024 | 32 | 128 | 64 | 2 |  |  |  |
| 1 | 512 | 32 | 32 | 16 | 1 | Ser83Ile | - | Val357Ile, His358Tyr, Arg541Lys, Asp564Lys |
| 1 | 512 | 32 | 128 | 64 | 1 | Ser83Ile | Ile390Thr, Val586Ala, Val799Ala, Ile811Val | Val357Ile, His358Tyr, Arg541Lys |
| 2 | 512 | 16 | 32 | 16 | 1 | Ser83Ile | Ile390Thr, Val586Ala, Val799Ala, Ile811Val | Val357Ile, His358Tyr, Arg541Lys, Asp564Lys |
|  | 512 | 32 | 128 | 64 | 1 |  |  |  |
| 1 | 512 | 16 | 64 | 16 | 1 | Ser83Ile | Gly752Val, Val768Ala, Val799Ala, Ile811Val, Glu827Asp, Met833Ile | Val357Ile, His358Tyr, Arg541Lys |
| 44 | 256 | 8 | 32 | 8 | 1 | Ser83Ile | Val799Ala, Ile811Val | Val357Ile, His358Tyr, Arg541Lys |
|  | 256 | 16 | 16 | 8 | 1 |  |  |  |
|  | 256 | 16 | 32 | 16 | 2 |  |  |  |
|  | 256 | 32 | 32 | 16 | 1 |  |  |  |
|  | 256 | 32 | 64 | 64 | 1 |  |  |  |
|  | 512 | 16 | 16 | 16 | 1 |  |  |  |
|  | 512 | 16 | 32 | 16 | 1 |  |  |  |
|  | 512 | 16 | 64 | 16 | 1 |  |  |  |
|  | 512 | 32 | 32 | 16 | 1 |  |  |  |
|  | 512 | 32 | 64 | 16 | 1 |  |  |  |
|  | 512 | 32 | 64 | 32 | 8 |  |  |  |
|  | 512 | 32 | 64 | 64 | 1 |  |  |  |
|  | 512 | 32 | 128 | 32 | 5 |  |  |  |
|  | 512 | 32 | 128 | 64 | 1 |  |  |  |
|  | 512 | 32 | 128 | 128 | 1 |  |  |  |
|  | 512 | 64 | 128 | 128 | 4 |  |  |  |
|  | 1024 | 16 | 32 | 16 | 2 |  |  |  |
|  | 1024 | 16 | 64 | 16 | 1 |  |  |  |
|  | 1024 | 32 | 32 | 32 | 1 |  |  |  |
|  | 1024 | 32 | 64 | 16 | 2 |  |  |  |
|  | 1024 | 32 | 64 | 64 | 1 |  |  |  |
|  | 1024 | 32 | 128 | 32 | 1 |  |  |  |
|  | 1024 | 32 | 128 | 64 | 2 |  |  |  |
|  | 1024 | 64 | 64 | 64 | 1 |  |  |  |
|  | 1024 | 64 | 128 | 128 | 2 |  |  |  |
| 17 | 256 | 32 | 32 | 32 | 1 | Ser83Ile | Ile390Thr, Val586Ala, Gly752Val, Val768Ala, Val799Ala, Ile811Val, Glu827Asp, Met833Il | Val357Ile, His358Tyr, Arg541Lys, Asp564Lys |
|  | 512 | 16 | 32 | 16 | 1 |  |  |  |
|  | 512 | 32 | 64 | 32 | 3 |  |  |  |
|  | 512 | 32 | 64 | 64 | 5 |  |  |  |
|  | 512 | 32 | 64 | 128 | 2 |  |  |  |
|  | 512 | 64 | 16 | 64 | 1 |  |  |  |
|  | 1024 | 32 | 64 | 32 | 2 |  |  |  |
|  | 1024 | 32 | 64 | 64 | 2 |  |  |  |
| 1 | 512 | 32 | 32 | 128 | 1 | Ser83Ile, Asn202Glu, Cys465Arg | - | Asp564Lys |
| 1 | 512 | 8 | 8 | 32 | 1 | Ser83Ile, Asn202Glu, Cys465Arg | Val799Ala, Ile811Val | Asp564Lys |
| 5 | 256 | 16 | 32 | 128 | 1 | Ser83Ile, Asn202Glu, Cys465Arg | Val586Thr, Val799Ala, Ile811Val | Asp564Lys |
|  | 512 | 8 | 8 | 8 | 1 |  |  |  |
|  | 512 | 16 | 32 | 32 | 1 |  |  |  |
|  | 512 | 32 | 32 | 128 | 2 |  |  |  |
| 1 | 512 | 16 | 16 | 128 | 1 | Ser83Ile, Asn202Glu, Cys465Arg | Val586Ala, Val799Ala, Ile811Val | Asp564Lys |
| 1 | 256 | 16 | 32 | 16 | 1 | Ser83Ile, Asn202Glu, Cys465Arg | Ile390Thr, Val586Ala, Val799Ala, Ile811Val | Arg541Lys, Asp564Lys |
| 1 | 512 | 16 | 32 | 16 | 1 | Ser83Ile, Asn202Glu, Cys465Arg | Ile390Thr, Val586Ala, Val799Ala, Ile811Val | Val357Ile, His358Tyr, Arg541Lys, Asp564Lys |
| 15 | 256 | 4 | 4 | 8 | 1 | Ser83Ile, Asn202Glu, Cys465Arg | Ile390Thr, Val586Ala, Gly752Val, Val768Ala, Val799Ala, Ile811Val, Glu827Asp, Met833Ile | Val357Ile, His358Tyr, Arg541Lys, Asp564Lys |
|  | 256 | 16 | 16 | 16 | 4 |  |  |  |
|  | 256 | 64 | 8 | 16 | 1 |  |  |  |
|  | 512 | 8 | 16 | 16 | 2 |  |  |  |
|  | 512 | 16 | 16 | 16 | 7 |  |  |  |
| 1 | 512 | 8 | 8 | 32 | 1 | Ser83Ile, Asn202Glu, Cys465Arg | Val799Ala, Ile811Val | Asp564Lys |
| 3 | 512 | 32 | 128 | 64 | 1 | Ser83Ile, Asn202Glu, Cys465Arg | Val799Ala, Ile811Val | Val357Ile, His358Tyr, Arg541Lys |
|  | 1024 | 64 | 128 | 64 | 1 |  |  |  |
|  | 1024 | 64 | 128 | 128 | 1 |  |  |  |
| 8 | 256 | 16 | 8 | 8 | 1 | Ser83Ile, Asn202Glu, Cys465Arg | Val586Ala, Val799Ala | Asp564Lys |
|  | 512 | 16 | 16 | 8 | 1 |  |  |  |
|  | 512 | 16 | 16 | 16 | 2 |  |  |  |
|  | 512 | 16 | 32 | 16 | 2 |  |  |  |
|  | 1024 | 32 | 64 | 16 | 2 |  |  |  |
| 2 | 512 | 32 | 32 | 32 | 1 | Ser83Arg | - | Val357Ile, His358Tyr, Arg541Lys |
|  | 512 | 64 | 64 | 8 | 1 |  |  |  |
| 15 | 512 | 8 | 16 | 8 | 1 | Ser83Arg, Cys465Arg | - | Val357Ile, His358Tyr, Arg541Lys |
|  | 512 | 16 | 16 | 8 | 1 |  |  |  |
|  | 512 | 16 | 16 | 16 | 3 |  |  |  |
|  | 512 | 16 | 32 | 16 | 1 |  |  |  |
|  | 512 | 32 | 16 | 16 | 4 |  |  |  |
|  | 512 | 32 | 32 | 8 | 3 |  |  |  |
|  | 512 | 64 | 64 | 64 | 1 |  |  |  |
|  | 512 | 64 | 128 | 128 | 1 |  |  |  |
| 1 | 512 | 32 | 32 | 32 | 1 | Ser83Arg, Cys465Arg | Ile390Thr, Val586Ala, Gly752Val, Val768Ala, Val799Ala, Ile811Val, Glu827Asp, Met833Ile | Val357Ile, His358Tyr, Arg541Lys, Asp564Lys |
| 2 | 252 | 2 | 4 | 2 | 1 | Ser83Arg, Asn202Glu, Cys465Arg | Val799Ala, Ile811Val | Asp564Lys |
|  | 252 | 4 | 4 | 4 | 1 |  |  |  |
| 5 | 256 | 2 | 4 | 2 | 1 | Ser83Arg, Asn202Glu, Cys465Arg | Val586Thr, Val799Ala, Ile811Val | Asp564Lys |
|  | 256 | 4 | 4 | 4 | 2 |  |  |  |
|  | 512 | 4 | 4 | 4 | 2 |  |  |  |
| 1 | 256 | 32 | 64 | 64 | 1 | Ser83Arg, Asn202Glu, Cys465Arg | Ile390Thr, Val586Ala, Gly752Val, Val768Ala, Val799Ala, Ile811Val, Glu827Asp, Met833Ile | Val357Ile, His358Tyr, Arg541Lys, Asp564Lys |

^a^NA, nalidixic acid; CIP, ciprofloxacin; ENR, enrofloxacin; OFX, ofloxacin.
