# Supplementary material for: Prevalence of fluoroquinolone resistance and mutations in the gyrA, parC and parE genes of Riemerella anatipestifer isolated from ducks in China
Source: BMC Microbiol. 2019 Dec 3;19:271. doi: 10.1186/s12866-019-1659-4 (PMC6892153; doi:10.1186/s12866-019-1659-4)
Supplement: Supplementary file 7 [file 12866_2019_1659_MOESM6_ESM.docx]

## Sheet: Sequence Data

Domain: Data  
							3	3	3	3	3	3			5	5	5	5			5	5	5
 	9	9	9	9			5	5	5	5	6	6			4	4	4	4			6	6	6
1	1	2	3	4			6	7	8	9	0	1			0	1	2	3			4	5	6
ATCC11845_Topoisomerase_IV_subunit_B	K	V	V	D			E	A	V	H	K	K			R	I	R	A			P	D	E
CCUG18373_Topoisomerase_IV_subunit_B	.	.	.	.			.	.	.	.	.	.			.	.	.	.			.	.	.
CCUG25001_Topoisomerase_IV_subunit_B	.	.	.	.			.	.	I	Y	.	.			.	.	K	.			.	E	.
CCUG25002_Topoisomerase_IV_subunit_B	.	.	.	.			.	.	I	Y	.	.			.	.	K	.			.	E	.
CCUG25004_Topoisomerase_IV_subunit_B	.	.	.	.			.	.	I	Y	.	.			.	.	K	.			.	E	.
CCUG25005_Topoisomerase_IV_subunit_B	.	.	.	.			.	.	I	Y	.	.			.	.	K	.			.	E	.
CCUG25008_Topoisomerase_IV_subunit_B	.	.	.	.			.	.	I	Y	.	.			.	.	K	.			.	E	.
CCUG25010_Topoisomerase_IV_subunit_B	.	.	.	.			.	.	I	Y	.	.			.	.	K	.			.	E	.
CCUG25054_Topoisomerase_IV_subunit_B	.	.	.	.			.	.	.	.	.	.			.	.	K	.			.	E	.
CCUG25055_Topoisomerase_IV_subunit_B	.	.	.	.			.	.	I	Y	.	.			.	.	K	.			.	E	.
RA-CH-1_Topoisomerase_IV_subunit_B	.	.	E	.			.	.	.	.	.	.			.	.	.	.			.	E	.
RA-CH-2_Topoisomerase_IV_subunit_B	.	.	.	.			.	.	I	Y	.	.			.	.	K	.			.	.	.
RCAD0111_Topoisomerase_IV_subunit_B	.	.	E	.			.	.	.	.	.	.			.	.	.	.			.	E	.
RCAD0121_Topoisomerase_IV_subunit_B	.	.	E	.			.	.	.	.	.	.			.	.	.	.			.	E	.
RCAD0122_Topoisomerase_IV_subunit_B	.	.	.	.			.	.	I	Y	.	.			.	.	K	.			.	E	.
RCAD0123_Topoisomerase_IV_subunit_B	.	.	.	.			.	.	I	Y	.	.			.	.	K	.			.	.	.
RCAD0124_Topoisomerase_IV_subunit_B	.	.	.	.			.	.	I	Y	.	.			.	.	K	.			.	.	.
RCAD0125_Topoisomerase_IV_subunit_B	.	.	.	.			.	.	I	Y	.	.			.	.	K	.			.	E	.
RCAD0127_Topoisomerase_IV_subunit_B	.	.	.	.			.	.	I	Y	.	.			.	.	K	.			.	.	.
RCAD0131_Topoisomerase_IV_subunit_B	.	.	.	.			.	.	I	Y	.	.			.	.	K	.			.	.	.
RCAD0133_Topoisomerase_IV_subunit_B	.	.	.	.			.	.	I	Y	.	.			.	.	K	.			.	E	.
RCAD0134_Topoisomerase_IV_subunit_B	.	.	.	.			.	.	I	Y	.	.			.	.	K	.			.	.	.
RCAD0135_Topoisomerase_IV_subunit_B	.	.	E	.			.	.	.	.	.	.			.	.	.	.			.	E	.
RCAD0142_Topoisomerase_IV_subunit_B	.	.	.	.			.	.	I	Y	.	.			.	.	K	.			.	.	.
RCAD0147_Topoisomerase_IV_subunit_B	.	.	.	.			.	.	I	Y	.	.			.	.	K	.			.	.	.
RCAD0150_Topoisomerase_IV_subunit_B	.	.	.	.			.	.	I	Y	.	.			.	.	K	.			.	.	.
RCAD0152-1_Topoisomerase_IV_subunit_B	.	.	E	.			.	.	.	.	.	.			.	.	.	.			.	E	.
RCAD0179_Topoisomerase_IV_subunit_B	.	.	.	.			.	.	.	.	.	.			.	.	.	.			.	.	.
RCAD0181_Topoisomerase_IV_subunit_B	.	.	.	.			.	.	I	Y	.	.			.	.	K	.			.	.	.
RCAD0183_Topoisomerase_IV_subunit_B	.	.	.	.			.	.	I	Y	.	.			.	.	K	.			.	.	.
RCAD0188_Topoisomerase_IV_subunit_B	.	.	.	.			.	.	I	Y	.	.			.	.	K	.			.	.	.
RCAD0282_Topoisomerase_IV_subunit_B	.	.	E	.			.	.	.	.	.	.			.	.	.	.			.	E	.
RCAD0377_Topoisomerase_IV_subunit_B	.	.	.	.			.	.	I	Y	.	.			.	.	K	.			.	E	.
RCAD0414_Topoisomerase_IV_subunit_B	.	.	.	.			.	.	I	Y	.	.			.	.	K	.			.	E	.
RCAD0421_Topoisomerase_IV_subunit_B	.	M	.	.			.	.	.	.	.	.			.	V	.	.			.	E	.
RCAD0422_Topoisomerase_IV_subunit_B	.	M	.	.			.	.	.	.	.	.			.	V	.	.			.	E	.
RCAD0427_Topoisomerase_IV_subunit_B	.	.	E	.			.	.	.	.	.	.			.	.	.	.			.	E	.

## Sheet: Info

Title	Comparative of ParE
Format
DataType	Protein
37
Number Sites	619
Identical	.
Missing	?
Indel	-
